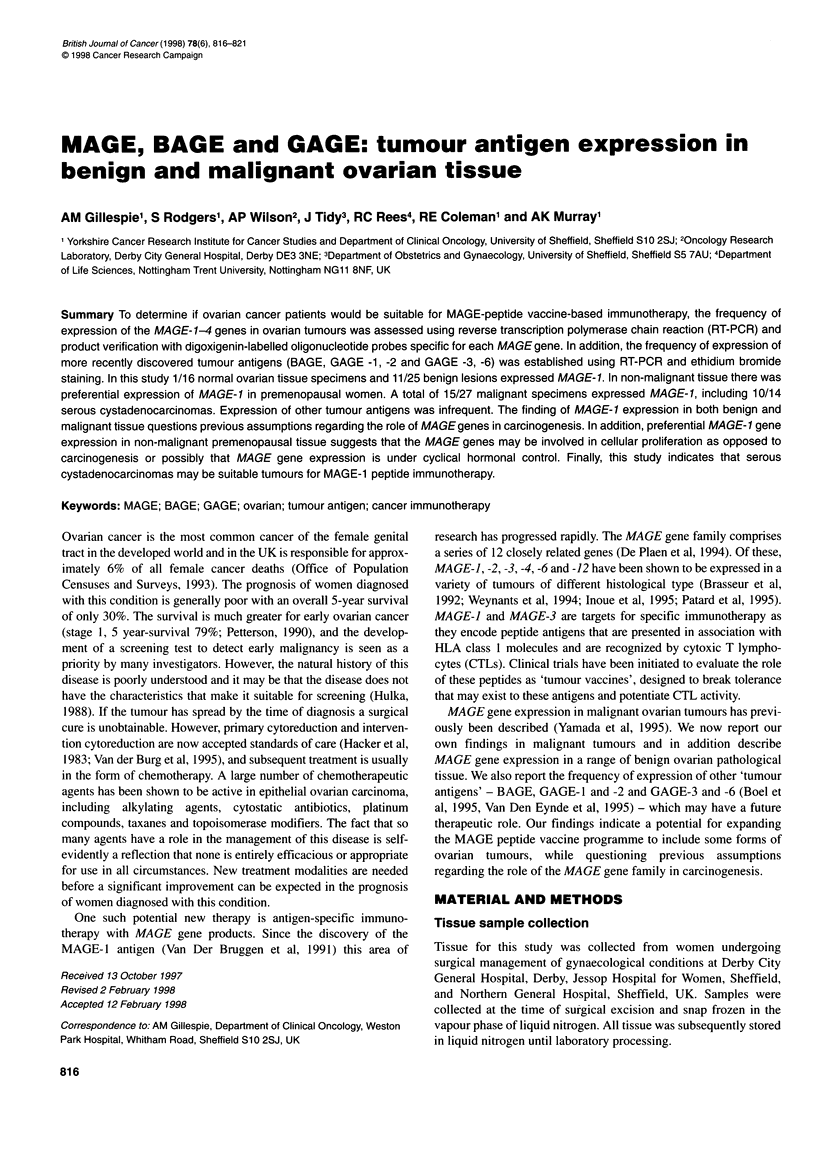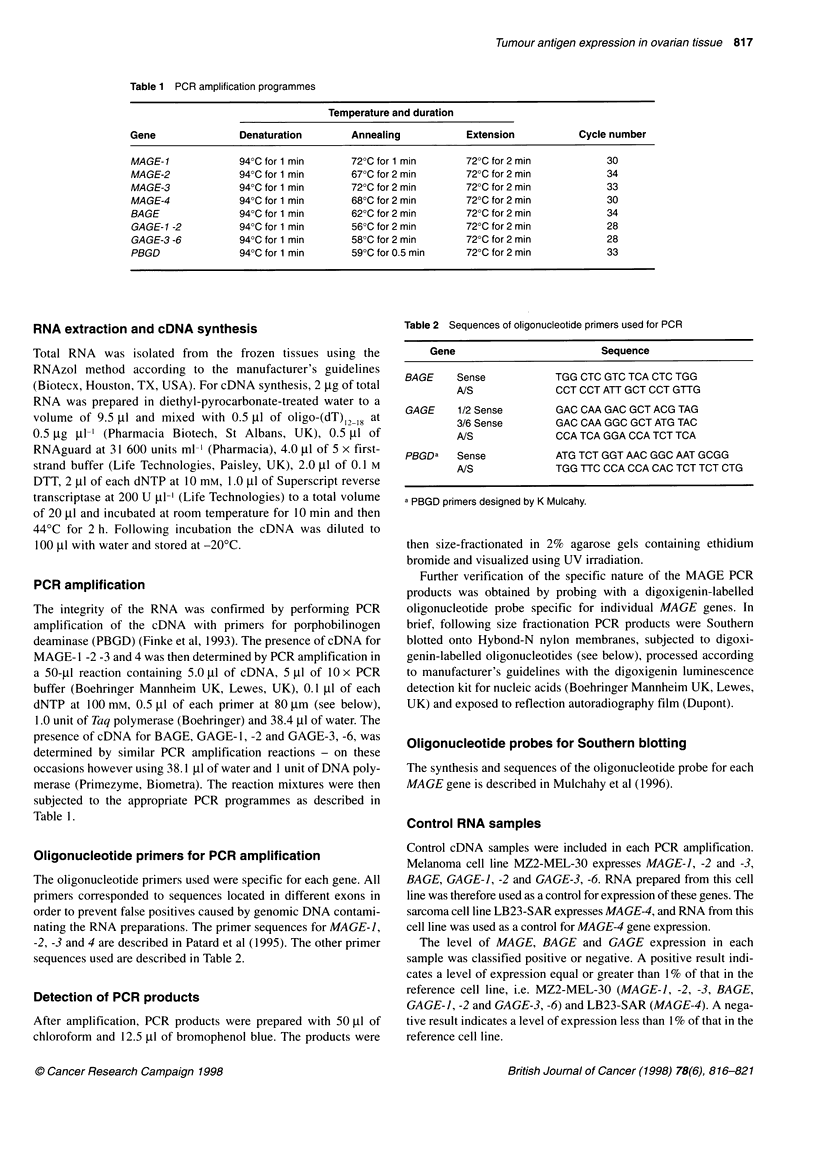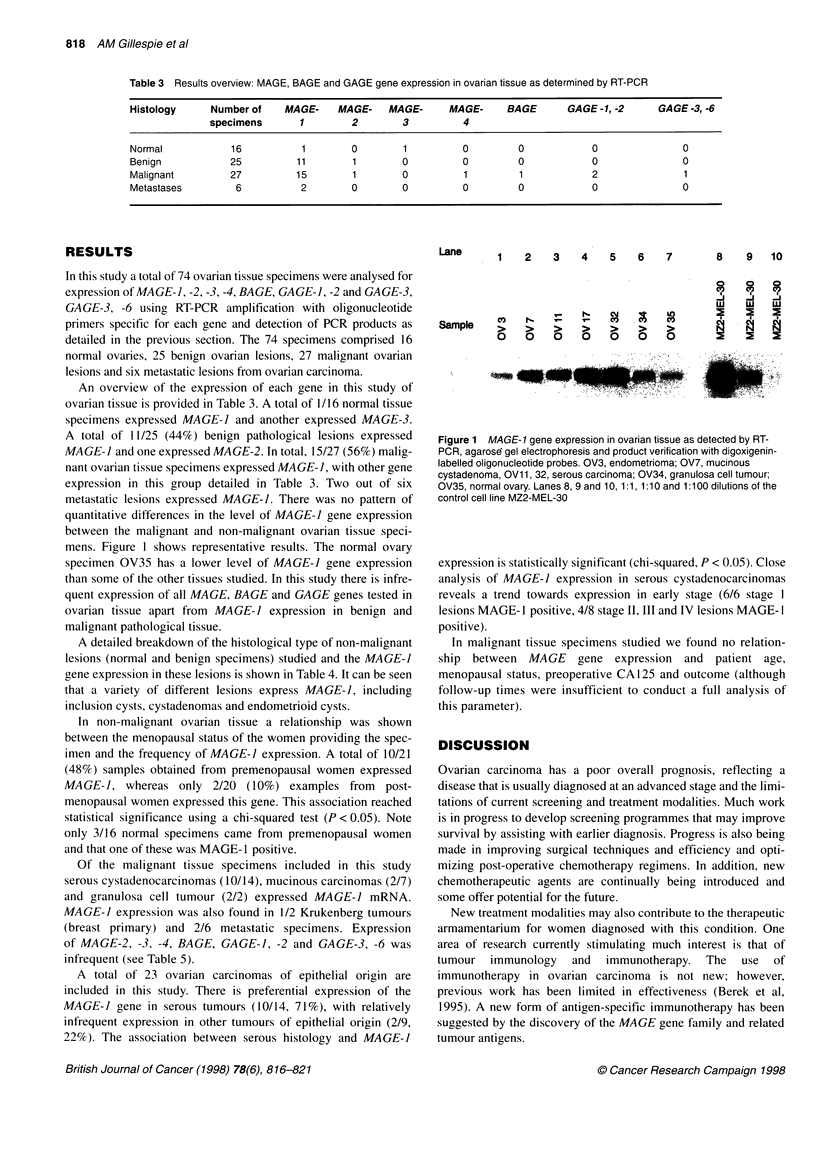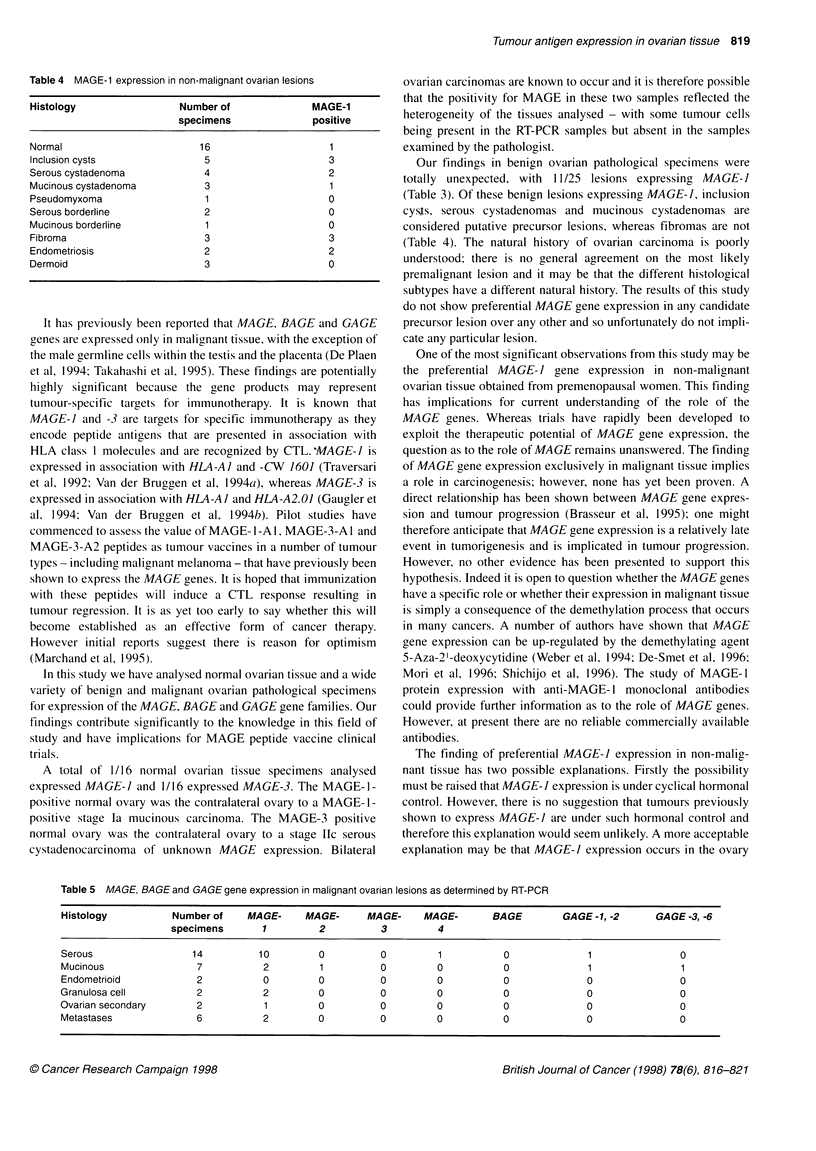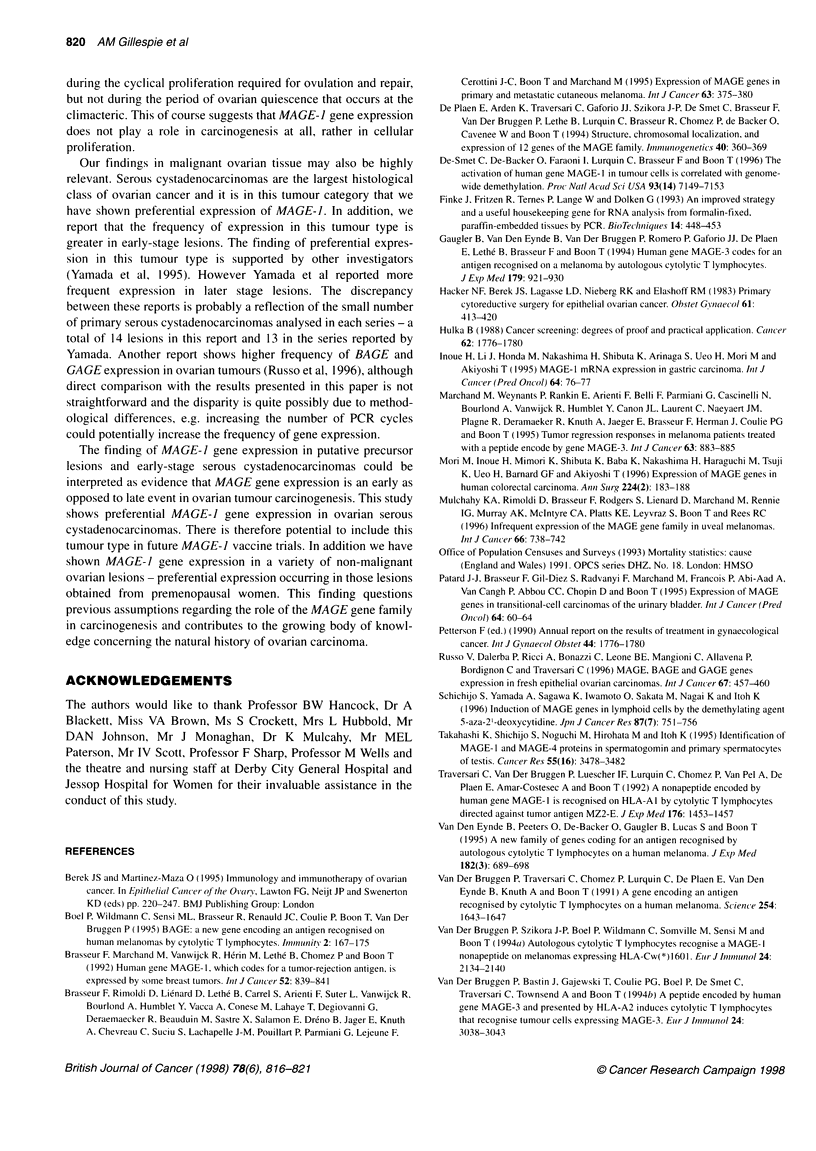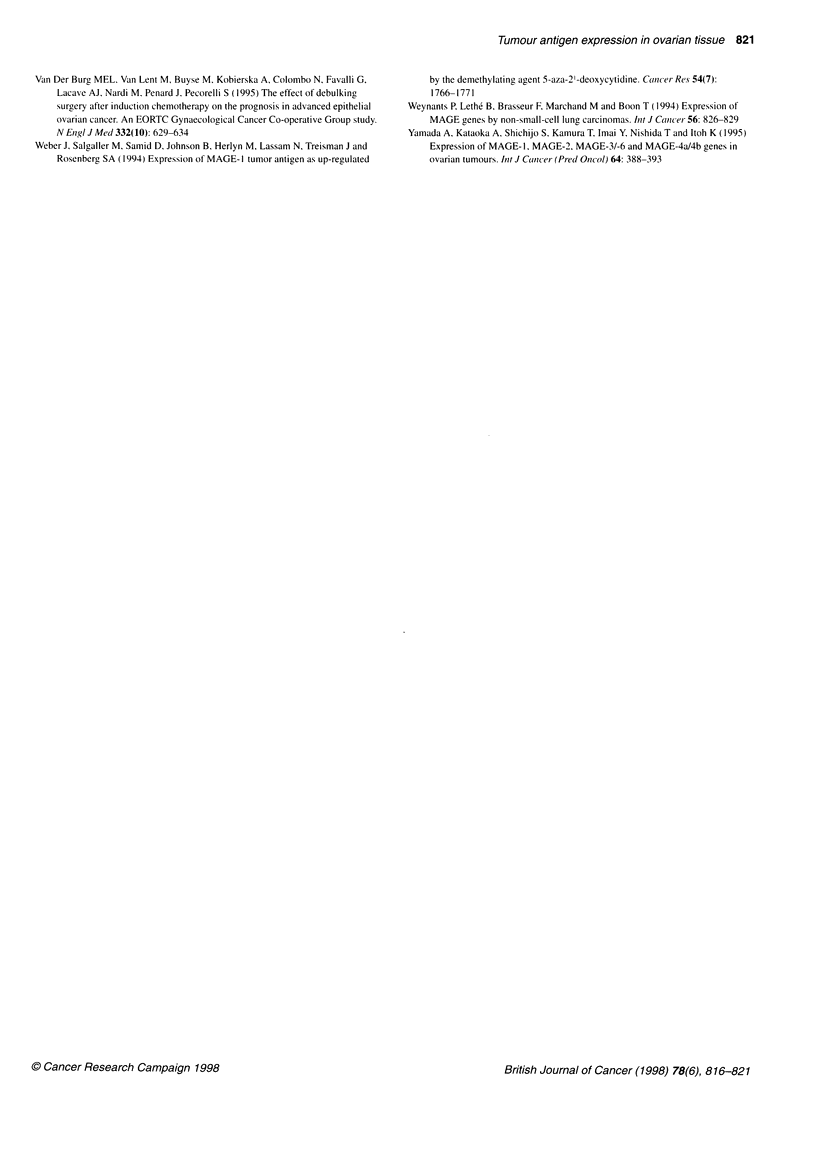# MAGE, BAGE and GAGE: tumour antigen expression in benign and malignant ovarian tissue.

**DOI:** 10.1038/bjc.1998.585

**Published:** 1998-09

**Authors:** A. M. Gillespie, S. Rodgers, A. P. Wilson, J. Tidy, R. C. Rees, R. E. Coleman, A. K. Murray

**Affiliations:** Yorkshire Cancer Research Institute for Cancer Studies and Department of Clinical Oncology, University of Sheffield, UK.

## Abstract

**Images:**


British Journal of Cancer (1998) 78(6), 816–821
© 1998 Cancer Research Campaign

### MAGE, BAGE and GAGE: tumour antigen expression in benign and malignant ovarian tissue

**AM Gillespie[1], S Rodgers[1], AP Wilson[2], J Tidy[3], RC Rees[4], RE Coleman[1] and AK Murray[1]**

[1] Yorkshire Cancer Research Institute for Cancer Studies and Department of Clinical Oncology, University of Sheffield, Sheffield S10 2SJ; [2]Oncology Research Laboratory, Derby City General Hospital, Derby DE3 3NE; [3]Department of Obstetrics and Gynaecology, University of Sheffield, Sheffield S5 7AU; [4]Department of Life Sciences, Nottingham Trent University, Nottingham NG11 8NF, UK

**Summary** To determine if ovarian cancer patients would be suitable for MAGE-peptide vaccine-based immunotherapy, the frequency of expression of the *MAGE-1–4* genes in ovarian tumours was assessed using reverse transcription polymerase chain reaction (RT-PCR) and product verification with digoxigenin-labelled oligonucleotide probes specific for each *MAGE* gene. In addition, the frequency of expression of more recently discovered tumour antigens (BAGE, GAGE -1, -2 and GAGE -3, -6) was established using RT-PCR and ethidium bromide staining. In this study 1/16 normal ovarian tissue specimens and 11/25 benign lesions expressed *MAGE-1*. In non-malignant tissue there was preferential expression of *MAGE-1* in premenopausal women. A total of 15/27 malignant specimens expressed *MAGE-1*, including 10/14 serous cystadenocarcinomas. Expression of other tumour antigens was infrequent. The finding of *MAGE-1* expression in both benign and malignant tissue questions previous assumptions regarding the role of *MAGE* genes in carcinogenesis. In addition, preferential *MAGE-1* gene expression in non-malignant premenopausal tissue suggests that the *MAGE* genes may be involved in cellular proliferation as opposed to carcinogenesis or possibly that *MAGE* gene expression is under cyclical hormonal control. Finally, this study indicates that serous cystadenocarcinomas may be suitable tumours for MAGE-1 peptide immunotherapy.

**Keywords:** MAGE; BAGE; GAGE; ovarian; tumour antigen; cancer immunotherapy

Ovarian cancer is the most common cancer of the female genital tract in the developed world and in the UK is responsible for approximately 6% of all female cancer deaths (Office of Population Censuses and Surveys, 1993). The prognosis of women diagnosed with this condition is generally poor with an overall 5-year survival of only 30%. The survival is much greater for early ovarian cancer (stage 1, 5 year-survival 79%; Petterson, 1990), and the development of a screening test to detect early malignancy is seen as a priority by many investigators. However, the natural history of this disease is poorly understood and it may be that the disease does not have the characteristics that make it suitable for screening (Hulka, 1988). If the tumour has spread by the time of diagnosis a surgical cure is unobtainable. However, primary cytoreduction and intervention cytoreduction are now accepted standards of care (Hacker et al, 1983; Van der Burg et al, 1995), and subsequent treatment is usually in the form of chemotherapy. A large number of chemotherapeutic agents has been shown to be active in epithelial ovarian carcinoma, including alkylating agents, cytostatic antibiotics, platinum compounds, taxanes and topoisomerase modifiers. The fact that so many agents have a role in the management of this disease is self-evidently a reflection that none is entirely efficacious or appropriate for use in all circumstances. New treatment modalities are needed before a significant improvement can be expected in the prognosis of women diagnosed with this condition.

One such potential new therapy is antigen-specific immunotherapy with *MAGE* gene products. Since the discovery of the MAGE-1 antigen (Van Der Bruggen et al, 1991) this area of research has progressed rapidly. The *MAGE* gene family comprises a series of 12 closely related genes (De Plaen et al, 1994). Of these, *MAGE-1*, *-2*, *-3*, *-4*, *-6* and *-12* have been shown to be expressed in a variety of tumours of different histological type (Brasseur et al, 1992; Weynants et al, 1994; Inoue et al, 1995; Patard et al, 1995). *MAGE-1* and *MAGE-3* are targets for specific immunotherapy as they encode peptide antigens that are presented in association with HLA class 1 molecules and are recognized by cytoxic T lymphocytes (CTLs). Clinical trials have been initiated to evaluate the role of these peptides as 'tumour vaccines', designed to break tolerance that may exist to these antigens and potentiate CTL activity.

*MAGE* gene expression in malignant ovarian tumours has previously been described (Yamada et al, 1995). We now report our own findings in malignant tumours and in addition describe *MAGE* gene expression in a range of benign ovarian pathological tissue. We also report the frequency of expression of other 'tumour antigens' – BAGE, GAGE-1 and -2 and GAGE-3 and -6 (Boel et al, 1995, Van Den Eynde et al, 1995) – which may have a future therapeutic role. Our findings indicate a potential for expanding the MAGE peptide vaccine programme to include some forms of ovarian tumours, while questioning previous assumptions regarding the role of the *MAGE* gene family in carcinogenesis.

### MATERIAL AND METHODS

### Tissue sample collection

Tissue for this study was collected from women undergoing surgical management of gynaecological conditions at Derby City General Hospital, Derby, Jessop Hospital for Women, Sheffield, and Northern General Hospital, Sheffield, UK. Samples were collected at the time of surgical excision and snap frozen in the vapour phase of liquid nitrogen. All tissue was subsequently stored in liquid nitrogen until laboratory processing.

*Received 13 October 1997*
*Revised 2 February 1998*
*Accepted 12 February 1998*

*Correspondence to:* AM Gillespie, Department of Clinical Oncology, Weston Park Hospital, Whitham Road, Sheffield S10 2SJ, UK

**Table 1** PCR amplification programmes

| Gene | Temperature and duration | | | Cycle number |
|---|---|---|---|---|
| | Denaturation | Annealing | Extension | |
| *MAGE-1* | 94°C for 1 min | 72°C for 1 min | 72°C for 2 min | 30 |
| *MAGE-2* | 94°C for 1 min | 67°C for 2 min | 72°C for 2 min | 34 |
| *MAGE-3* | 94°C for 1 min | 72°C for 2 min | 72°C for 2 min | 33 |
| *MAGE-4* | 94°C for 1 min | 68°C for 2 min | 72°C for 2 min | 30 |
| *BAGE* | 94°C for 1 min | 62°C for 2 min | 72°C for 2 min | 34 |
| *GAGE-1 -2* | 94°C for 1 min | 56°C for 2 min | 72°C for 2 min | 28 |
| *GAGE-3 -6* | 94°C for 1 min | 58°C for 2 min | 72°C for 2 min | 28 |
| *PBGD* | 94°C for 1 min | 59°C for 0.5 min | 72°C for 2 min | 33 |

### RNA extraction and cDNA synthesis

Total RNA was isolated from the frozen tissues using the RNAzol method according to the manufacturer's guidelines (Biotecx, Houston, TX, USA). For cDNA synthesis, 2 μg of total RNA was prepared in diethyl-pyrocarbonate-treated water to a volume of 9.5 μl and mixed with 0.5 μl of oligo-$(dT)_{12-18}$ at 0.5 μg $\mu l^{-1}$ (Pharmacia Biotech, St Albans, UK), 0.5 μl of RNAguard at 31 600 units $ml^{-1}$ (Pharmacia), 4.0 μl of 5 × first-strand buffer (Life Technologies, Paisley, UK), 2.0 μl of 0.1 M DTT, 2 μl of each dNTP at 10 mM, 1.0 μl of Superscript reverse transcriptase at 200 U $\mu l^{-1}$ (Life Technologies) to a total volume of 20 μl and incubated at room temperature for 10 min and then 44°C for 2 h. Following incubation the cDNA was diluted to 100 μl with water and stored at –20°C.

### PCR amplification

The integrity of the RNA was confirmed by performing PCR amplification of the cDNA with primers for porphobilinogen deaminase (PBGD) (Finke et al, 1993). The presence of cDNA for MAGE-1 -2 -3 and 4 was then determined by PCR amplification in a 50-μl reaction containing 5.0 μl of cDNA, 5 μl of 10 × PCR buffer (Boehringer Mannheim UK, Lewes, UK), 0.1 μl of each dNTP at 100 mM, 0.5 μl of each primer at 80 μm (see below), 1.0 unit of *Taq* polymerase (Boehringer) and 38.4 μl of water. The presence of cDNA for BAGE, GAGE-1, -2 and GAGE-3, -6, was determined by similar PCR amplification reactions – on these occasions however using 38.1 μl of water and 1 unit of DNA polymerase (Primezyme, Biometra). The reaction mixtures were then subjected to the appropriate PCR programmes as described in Table 1.

### Oligonucleotide primers for PCR amplification

The oligonucleotide primers used were specific for each gene. All primers corresponded to sequences located in different exons in order to prevent false positives caused by genomic DNA contaminating the RNA preparations. The primer sequences for *MAGE-1*, *-2*, *-3* and *4* are described in Patard et al (1995). The other primer sequences used are described in Table 2.

### Detection of PCR products

After amplification, PCR products were prepared with 50 μl of chloroform and 12.5 μl of bromophenol blue. The products were then size-fractionated in 2% agarose gels containing ethidium bromide and visualized using UV irradiation.

**Table 2** Sequences of oligonucleotide primers used for PCR

| Gene | | Sequence |
|---|---|---|
| *BAGE* | Sense | TGG CTC GTC TCA CTC TGG |
| | A/S | CCT CCT ATT GCT CCT GTTG |
| *GAGE* | 1/2 Sense | GAC CAA GAC GCT ACG TAG |
| | 3/6 Sense | GAC CAA GGC GCT ATG TAC |
| | A/S | CCA TCA GGA CCA TCT TCA |
| *PBGD*[a] | Sense | ATG TCT GGT AAC GGC AAT GCGG |
| | A/S | TGG TTC CCA CCA CAC TCT TCT CTG |

[a] PBGD primers designed by K Mulcahy.

Further verification of the specific nature of the MAGE PCR products was obtained by probing with a digoxigenin-labelled oligonucleotide probe specific for individual *MAGE* genes. In brief, following size fractionation PCR products were Southern blotted onto Hybond-N nylon membranes, subjected to digoxigenin-labelled oligonucleotides (see below), processed according to manufacturer's guidelines with the digoxigenin luminescence detection kit for nucleic acids (Boehringer Mannheim UK, Lewes, UK) and exposed to reflection autoradiography film (Dupont).

### Oligonucleotide probes for Southern blotting

The synthesis and sequences of the oligonucleotide probe for each *MAGE* gene is described in Mulchahy et al (1996).

### Control RNA samples

Control cDNA samples were included in each PCR amplification. Melanoma cell line MZ2-MEL-30 expresses *MAGE-1*, *-2* and *-3*, *BAGE*, *GAGE-1*, *-2* and *GAGE-3*, *-6*. RNA prepared from this cell line was therefore used as a control for expression of these genes. The sarcoma cell line LB23-SAR expresses *MAGE-4*, and RNA from this cell line was used as a control for *MAGE-4* gene expression.

The level of *MAGE*, *BAGE* and *GAGE* expression in each sample was classified positive or negative. A positive result indicates a level of expression equal or greater than 1% of that in the reference cell line, i.e. MZ2-MEL-30 (*MAGE-1*, *-2*, *-3*, *BAGE*, *GAGE-1*, *-2* and *GAGE-3*, *-6*) and LB23-SAR (*MAGE-4*). A negative result indicates a level of expression less than 1% of that in the reference cell line.

© Cancer Research Campaign 1998

**Table 3** Results overview: MAGE, BAGE and GAGE gene expression in ovarian tissue as determined by RT-PCR

| Histology | Number of specimens | *MAGE-1* | *MAGE-2* | *MAGE-3* | *MAGE-4* | *BAGE* | *GAGE -1, -2* | *GAGE -3, -6* |
|---|---|---|---|---|---|---|---|---|
| Normal | 16 | 1 | 0 | 1 | 0 | 0 | 0 | 0 |
| Benign | 25 | 11 | 1 | 0 | 0 | 0 | 0 | 0 |
| Malignant | 27 | 15 | 1 | 0 | 1 | 1 | 2 | 1 |
| Metastases | 6 | 2 | 0 | 0 | 0 | 0 | 0 | 0 |

### RESULTS

In this study a total of 74 ovarian tissue specimens were analysed for expression of *MAGE-1*, *-2*, *-3*, *-4*, *BAGE*, *GAGE-1*, *-2* and *GAGE-3*, *GAGE-3*, *-6* using RT-PCR amplification with oligonucleotide primers specific for each gene and detection of PCR products as detailed in the previous section. The 74 specimens comprised 16 normal ovaries, 25 benign ovarian lesions, 27 malignant ovarian lesions and six metastatic lesions from ovarian carcinoma.

An overview of the expression of each gene in this study of ovarian tissue is provided in Table 3. A total of 1/16 normal tissue specimens expressed *MAGE-1* and another expressed *MAGE-3*. A total of 11/25 (44%) benign pathological lesions expressed *MAGE-1* and one expressed *MAGE-2*. In total, 15/27 (56%) malignant ovarian tissue specimens expressed *MAGE-1*, with other gene expression in this group detailed in Table 3. Two out of six metastatic lesions expressed *MAGE-1*. There was no pattern of quantitative differences in the level of *MAGE-1* gene expression between the malignant and non-malignant ovarian tissue specimens. Figure 1 shows representative results. The normal ovary specimen OV35 has a lower level of *MAGE-1* gene expression than some of the other tissues studied. In this study there is infrequent expression of all *MAGE*, *BAGE* and *GAGE* genes tested in ovarian tissue apart from *MAGE-1* expression in benign and malignant pathological tissue.

A detailed breakdown of the histological type of non-malignant lesions (normal and benign specimens) studied and the *MAGE-1* gene expression in these lesions is shown in Table 4. It can be seen that a variety of different lesions express *MAGE-1*, including inclusion cysts, cystadenomas and endometrioid cysts.

In non-malignant ovarian tissue a relationship was shown between the menopausal status of the women providing the specimen and the frequency of *MAGE-1* expression. A total of 10/21 (48%) samples obtained from premenopausal women expressed *MAGE-1*, whereas only 2/20 (10%) examples from postmenopausal women expressed this gene. This association reached statistical significance using a chi-squared test ($P < 0.05$). Note only 3/16 normal specimens came from premenopausal women and that one of these was MAGE-1 positive.

Of the malignant tissue specimens included in this study serous cystadenocarcinomas (10/14), mucinous carcinomas (2/7) and granulosa cell tumour (2/2) expressed *MAGE-1* mRNA. *MAGE-1* expression was also found in 1/2 Krukenberg tumours (breast primary) and 2/6 metastatic specimens. Expression of *MAGE-2*, *-3*, *-4*, *BAGE*, *GAGE-1*, *-2* and *GAGE-3*, *-6* was infrequent (see Table 5).

A total of 23 ovarian carcinomas of epithelial origin are included in this study. There is preferential expression of the *MAGE-1* gene in serous tumours (10/14, 71%), with relatively infrequent expression in other tumours of epithelial origin (2/9, 22%). The association between serous histology and *MAGE-1* expression is statistically significant (chi-squared, $P < 0.05$). Close analysis of *MAGE-1* expression in serous cystadenocarcinomas reveals a trend towards expression in early stage (6/6 stage 1 lesions MAGE-1 positive, 4/8 stage II, III and IV lesions MAGE-1 positive).

**Figure 1** *MAGE-1* gene expression in ovarian tissue as detected by RT-PCR, agarose gel electrophoresis and product verification with digoxigenin-labelled oligonucleotide probes. OV3, endometrioma; OV7, mucinous cystadenoma, OV11, 32, serous carcinoma; OV34, granulosa cell tumour; OV35, normal ovary. Lanes 8, 9 and 10, 1:1, 1:10 and 1:100 dilutions of the control cell line MZ2-MEL-30

In malignant tissue specimens studied we found no relationship between *MAGE* gene expression and patient age, menopausal status, preoperative CA125 and outcome (although follow-up times were insufficient to conduct a full analysis of this parameter).

### DISCUSSION

Ovarian carcinoma has a poor overall prognosis, reflecting a disease that is usually diagnosed at an advanced stage and the limitations of current screening and treatment modalities. Much work is in progress to develop screening programmes that may improve survival by assisting with earlier diagnosis. Progress is also being made in improving surgical techniques and efficiency and optimizing post-operative chemotherapy regimens. In addition, new chemotherapeutic agents are continually being introduced and some offer potential for the future.

New treatment modalities may also contribute to the therapeutic armamentarium for women diagnosed with this condition. One area of research currently stimulating much interest is that of tumour immunology and immunotherapy. The use of immunotherapy in ovarian carcinoma is not new; however, previous work has been limited in effectiveness (Berek et al, 1995). A new form of antigen-specific immunotherapy has been suggested by the discovery of the *MAGE* gene family and related tumour antigens.

© Cancer Research Campaign 1998

**Table 4** MAGE-1 expression in non-malignant ovarian lesions

| Histology | Number of specimens | MAGE-1 positive |
|---|---|---|
| Normal | 16 | 1 |
| Inclusion cysts | 5 | 3 |
| Serous cystadenoma | 4 | 2 |
| Mucinous cystadenoma | 3 | 1 |
| Pseudomyxoma | 1 | 0 |
| Serous borderline | 2 | 0 |
| Mucinous borderline | 1 | 0 |
| Fibroma | 3 | 3 |
| Endometriosis | 2 | 2 |
| Dermoid | 3 | 0 |

It has previously been reported that *MAGE*, *BAGE* and *GAGE* genes are expressed only in malignant tissue, with the exception of the male germline cells within the testis and the placenta (De Plaen et al, 1994; Takahashi et al, 1995). These findings are potentially highly significant because the gene products may represent tumour-specific targets for immunotherapy. It is known that *MAGE-1* and *-3* are targets for specific immunotherapy as they encode peptide antigens that are presented in association with HLA class 1 molecules and are recognized by CTL. *MAGE-1* is expressed in association with *HLA-A1* and *-CW 1601* (Traversari et al, 1992; Van der Bruggen et al, 1994*a*), whereas *MAGE-3* is expressed in association with *HLA-A1* and *HLA-A2.01* (Gaugler et al, 1994; Van der Bruggen et al, 1994*b*). Pilot studies have commenced to assess the value of MAGE-1-A1, MAGE-3-A1 and MAGE-3-A2 peptides as tumour vaccines in a number of tumour types – including malignant melanoma – that have previously been shown to express the *MAGE* genes. It is hoped that immunization with these peptides will induce a CTL response resulting in tumour regression. It is as yet too early to say whether this will become established as an effective form of cancer therapy. However initial reports suggest there is reason for optimism (Marchand et al, 1995).

In this study we have analysed normal ovarian tissue and a wide variety of benign and malignant ovarian pathological specimens for expression of the *MAGE*, *BAGE* and *GAGE* gene families. Our findings contribute significantly to the knowledge in this field of study and have implications for MAGE peptide vaccine clinical trials.

A total of 1/16 normal ovarian tissue specimens analysed expressed *MAGE-1* and 1/16 expressed *MAGE-3*. The MAGE-1-positive normal ovary was the contralateral ovary to a MAGE-1-positive stage Ia mucinous carcinoma. The MAGE-3 positive normal ovary was the contralateral ovary to a stage IIc serous cystadenocarcinoma of unknown *MAGE* expression. Bilateral ovarian carcinomas are known to occur and it is therefore possible that the positivity for MAGE in these two samples reflected the heterogeneity of the tissues analysed – with some tumour cells being present in the RT-PCR samples but absent in the samples examined by the pathologist.

Our findings in benign ovarian pathological specimens were totally unexpected, with 11/25 lesions expressing *MAGE-1* (Table 3). Of these benign lesions expressing *MAGE-1*, inclusion cysts, serous cystadenomas and mucinous cystadenomas are considered putative precursor lesions, whereas fibromas are not (Table 4). The natural history of ovarian carcinoma is poorly understood; there is no general agreement on the most likely premalignant lesion and it may be that the different histological subtypes have a different natural history. The results of this study do not show preferential *MAGE* gene expression in any candidate precursor lesion over any other and so unfortunately do not implicate any particular lesion.

One of the most significant observations from this study may be the preferential *MAGE-1* gene expression in non-malignant ovarian tissue obtained from premenopausal women. This finding has implications for current understanding of the role of the *MAGE* genes. Whereas trials have rapidly been developed to exploit the therapeutic potential of *MAGE* gene expression, the question as to the role of *MAGE* remains unanswered. The finding of *MAGE* gene expression exclusively in malignant tissue implies a role in carcinogenesis; however, none has yet been proven. A direct relationship has been shown between *MAGE* gene expression and tumour progression (Brasseur et al, 1995); one might therefore anticipate that *MAGE* gene expression is a relatively late event in tumorigenesis and is implicated in tumour progression. However, no other evidence has been presented to support this hypothesis. Indeed it is open to question whether the *MAGE* genes have a specific role or whether their expression in malignant tissue is simply a consequence of the demethylation process that occurs in many cancers. A number of authors have shown that *MAGE* gene expression can be up-regulated by the demethylating agent 5-Aza-2'-deoxycytidine (Weber et al, 1994; De-Smet et al, 1996; Mori et al, 1996; Shichijo et al, 1996). The study of MAGE-1 protein expression with anti-MAGE-1 monoclonal antibodies could provide further information as to the role of *MAGE* genes. However, at present there are no reliable commercially available antibodies.

The finding of preferential *MAGE-1* expression in non-malignant tissue has two possible explanations. Firstly the possibility must be raised that *MAGE-1* expression is under cyclical hormonal control. However, there is no suggestion that tumours previously shown to express *MAGE-1* are under such hormonal control and therefore this explanation would seem unlikely. A more acceptable explanation may be that *MAGE-1* expression occurs in the ovary

**Table 5** *MAGE*, *BAGE* and *GAGE* gene expression in malignant ovarian lesions as determined by RT-PCR

| Histology | Number of specimens | *MAGE-1* | *MAGE-2* | *MAGE-3* | *MAGE-4* | *BAGE* | *GAGE -1, -2* | *GAGE -3, -6* |
|---|---|---|---|---|---|---|---|---|
| Serous | 14 | 10 | 0 | 0 | 1 | 0 | 1 | 0 |
| Mucinous | 7 | 2 | 1 | 0 | 0 | 0 | 1 | 1 |
| Endometrioid | 2 | 0 | 0 | 0 | 0 | 0 | 0 | 0 |
| Granulosa cell | 2 | 2 | 0 | 0 | 0 | 0 | 0 | 0 |
| Ovarian secondary | 2 | 1 | 0 | 0 | 0 | 0 | 0 | 0 |
| Metastases | 6 | 2 | 0 | 0 | 0 | 0 | 0 | 0 |

© Cancer Research Campaign 1998

during the cyclical proliferation required for ovulation and repair, but not during the period of ovarian quiescence that occurs at the climacteric. This of course suggests that *MAGE-1* gene expression does not play a role in carcinogenesis at all, rather in cellular proliferation.

Our findings in malignant ovarian tissue may also be highly relevant. Serous cystadenocarcinomas are the largest histological class of ovarian cancer and it is in this tumour category that we have shown preferential expression of *MAGE-1*. In addition, we report that the frequency of expression in this tumour type is greater in early-stage lesions. The finding of preferential expression in this tumour type is supported by other investigators (Yamada et al, 1995). However Yamada et al reported more frequent expression in later stage lesions. The discrepancy between these reports is probably a reflection of the small number of primary serous cystadenocarcinomas analysed in each series – a total of 14 lesions in this report and 13 in the series reported by Yamada. Another report shows higher frequency of *BAGE* and *GAGE* expression in ovarian tumours (Russo et al, 1996), although direct comparison with the results presented in this paper is not straightforward and the disparity is quite possibly due to methodological differences, e.g. increasing the number of PCR cycles could potentially increase the frequency of gene expression.

The finding of *MAGE-1* gene expression in putative precursor lesions and early-stage serous cystadenocarcinomas could be interpreted as evidence that *MAGE* gene expression is an early as opposed to late event in ovarian tumour carcinogenesis. This study shows preferential *MAGE-1* gene expression in ovarian serous cystadenocarcinomas. There is therefore potential to include this tumour type in future *MAGE-1* vaccine trials. In addition we have shown *MAGE-1* gene expression in a variety of non-malignant ovarian lesions – preferential expression occurring in those lesions obtained from premenopausal women. This finding questions previous assumptions regarding the role of the *MAGE* gene family in carcinogenesis and contributes to the growing body of knowledge concerning the natural history of ovarian carcinoma.

### ACKNOWLEDGEMENTS

The authors would like to thank Professor BW Hancock, Dr A Blackett, Miss VA Brown, Ms S Crockett, Mrs L Hubbold, Mr DAN Johnson, Mr J Monaghan, Dr K Mulcahy, Mr MEL Paterson, Mr IV Scott, Professor F Sharp, Professor M Wells and the theatre and nursing staff at Derby City General Hospital and Jessop Hospital for Women for their invaluable assistance in the conduct of this study.

### REFERENCES

Berek JS and Martinez-Maza O (1995) Immunology and immunotherapy of ovarian cancer. In *Epithelial Cancer of the Ovary*, Lawton FG, Neijt JP and Swenerton KD (eds) pp. 220–247. BMJ Publishing Group: London

Boel P, Wildmann C, Sensi ML, Brasseur R, Renauld JC, Coulie P, Boon T, Van Der Bruggen P (1995) BAGE: a new gene encoding an antigen recognised on human melanomas by cytolytic T lymphocytes. *Immunity* **2**: 167–175

Brasseur F, Marchand M, Vanwijck R, Hérin M, Lethé B, Chomez P and Boon T (1992) Human gene MAGE-1, which codes for a tumor-rejection antigen, is expressed by some breast tumors. *Int J Cancer* **52**: 839–841

Brasseur F, Rimoldi D, Liénard D, Lethé B, Carrel S, Arienti F, Suter L, Vanwijck R, Bourlond A, Humblet Y, Vacca A, Conese M, Lahaye T, Degiovanni G, Deraemaecker R, Beauduin M, Sastre X, Salamon E, Dréno B, Jager E, Knuth A, Chevreau C, Suciu S, Lachapelle J-M, Pouillart P, Parmiani G, Lejeune F, Cerottini J-C, Boon T and Marchand M (1995) Expression of MAGE genes in primary and metastatic cutaneous melanoma. *Int J Cancer* **63**: 375–380

De Plaen E, Arden K, Traversari C, Gaforio JJ, Szikora J-P, De Smet C, Brasseur F, Van Der Bruggen P, Lethe B, Lurquin C, Brasseur R, Chomez P, de Backer O, Cavenee W and Boon T (1994) Structure, chromosomal localization, and expression of 12 genes of the MAGE family. *Immunogenetics* **40**: 360–369

De-Smet C, De-Backer O, Faraoni I, Lurquin C, Brasseur F and Boon T (1996) The activation of human gene MAGE-1 in tumour cells is correlated with genome-wide demethylation. *Proc Natl Acad Sci USA* **93(14)** 7149–7153

Finke J, Fritzen R, Ternes P, Lange W and Dolken G (1993) An improved strategy and a useful housekeeping gene for RNA analysis from formalin-fixed, paraffin-embedded tissues by PCR. *BioTechniques* **14**: 448–453

Gaugler B, Van Den Eynde B, Van Der Bruggen P, Romero P, Gaforio JJ, De Plaen E, Lethé B, Brasseur F and Boon T (1994) Human gene MAGE-3 codes for an antigen recognised on a melanoma by autologous cytolytic T lymphocytes. *J Exp Med* **179**: 921–930

Hacker NF, Berek JS, Lagasse LD, Nieberg RK and Elashoff RM (1983) Primary cytoreductive surgery for epithelial ovarian cancer. *Obstet Gynaecol* **61**: 413–420

Hulka B (1988) Cancer screening: degrees of proof and practical application. *Cancer* **62**: 1776–1780

Inoue H, Li J, Honda M, Nakashima H, Shibuta K, Arinaga S, Ueo H, Mori M and Akiyoshi T (1995) MAGE-1 mRNA expression in gastric carcinoma. *Int J Cancer (Pred Oncol)* **64**: 76–77

Marchand M, Weynants P, Rankin E, Arienti F, Belli F, Parmiani G, Cascinelli N, Bourlond A, Vanwijck R, Humblet Y, Canon JL, Laurent C, Naeyaert JM, Plagne R, Deramaeker R, Knuth A, Jaeger E, Brasseur F, Herman J, Coulie PG and Boon T (1995) Tumor regression responses in melanoma patients treated with a peptide encode by gene MAGE-3. *Int J Cancer* **63**: 883–885

Mori M, Inoue H, Mimori K, Shibuta K, Baba K, Nakashima H, Haraguchi M, Tsuji K, Ueo H, Barnard GF and Akiyoshi T (1996) Expression of MAGE genes in human colorectal carcinoma. *Ann Surg* **224(2)**: 183–188

Mulchahy KA, Rimoldi D, Brasseur F, Rodgers S, Lienard D, Marchand M, Rennie IG, Murray AK, McIntyre CA, Platts KE, Leyvraz S, Boon T and Rees RC (1996) Infrequent expression of the MAGE gene family in uveal melanomas. *Int J Cancer* **66**: 738–742

Office of Population Censuses and Surveys (1993) Mortality statistics: cause (England and Wales) 1991. OPCS series DHZ, No. 18. London: HMSO

Patard J-J, Brasseur F, Gil-Diez S, Radvanyi F, Marchand M, Francois P, Abi-Aad A, Van Cangh P, Abbou CC, Chopin D and Boon T (1995) Expression of MAGE genes in transitional-cell carcinomas of the urinary bladder. *Int J Cancer (Pred Oncol)* **64**: 60–64

Petterson F (ed.) (1990) Annual report on the results of treatment in gynaecological cancer. *Int J Gynaecol Obstet* **44**: 1776–1780

Russo V, Dalerba P, Ricci A, Bonazzi C, Leone BE, Mangioni C, Allavena P, Bordignon C and Traversari C (1996) MAGE, BAGE and GAGE genes expression in fresh epithelial ovarian carcinomas. *Int J Cancer* **67**: 457–460

Schichijo S, Yamada A, Sagawa K, Iwamoto O, Sakata M, Nagai K and Itoh K (1996) Induction of MAGE genes in lymphoid cells by the demethylating agent 5-aza-2'-deoxycytidine. *Jpn J Cancer Res* **87(7)**: 751–756

Takahashi K, Shichijo S, Noguchi M, Hirohata M and Itoh K (1995) Identification of MAGE-1 and MAGE-4 proteins in spermatogomin and primary spermatocytes of testis. *Cancer Res* **55(16)**: 3478–3482

Traversari C, Van Der Bruggen P, Luescher IF, Lurquin C, Chomez P, Van Pel A, De Plaen E, Amar-Costesec A and Boon T (1992) A nonapeptide encoded by human gene MAGE-1 is recognised on HLA-A1 by cytolytic T lymphocytes directed against tumor antigen MZ2-E. *J Exp Med* **176**: 1453–1457

Van Den Eynde B, Peeters O, De-Backer O, Gaugler B, Lucas S and Boon T (1995) A new family of genes coding for an antigen recognised by autologous cytolytic T lymphocytes on a human melanoma. *J Exp Med* **182(3)**: 689–698

Van Der Bruggen P, Traversari C, Chomez P, Lurquin C, De Plaen E, Van Den Eynde B, Knuth A and Boon T (1991) A gene encoding an antigen recognised by cytolytic T lymphocytes on a human melanoma. *Science* **254**: 1643–1647

Van Der Bruggen P, Szikora J-P, Boel P, Wildmann C, Somville M, Sensi M and Boon T (1994*a*) Autologous cytolytic T lymphocytes recognise a MAGE-1 nonapeptide on melanomas expressing HLA-Cw(*)1601. *Eur J Immunol* **24**: 2134–2140

Van Der Bruggen P, Bastin J, Gajewski T, Coulie PG, Boel P, De Smet C, Traversari C, Townsend A and Boon T (1994*b*) A peptide encoded by human gene MAGE-3 and presented by HLA-A2 induces cytolytic T lymphocytes that recognise tumour cells expressing MAGE-3. *Eur J Immunol* **24**: 3038–3043

© Cancer Research Campaign 1998

Van Der Burg MEL, Van Lent M, Buyse M, Kobierska A, Colombo N, Favalli G, Lacave AJ, Nardi M, Penard J, Pecorelli S (1995) The effect of debulking surgery after induction chemotherapy on the prognosis in advanced epithelial ovarian cancer. An EORTC Gynaecological Cancer Co-operative Group study. *N Engl J Med* **332(10)**: 629–634

Weber J, Salgaller M, Samid D, Johnson B, Herlyn M, Lassam N, Treisman J and Rosenberg SA (1994) Expression of MAGE-1 tumor antigen as up-regulated by the demethylating agent 5-aza-2'-deoxycytidine. *Cancer Res* **54(7)**: 1766–1771

Weynants P, Lethé B, Brasseur F, Marchand M and Boon T (1994) Expression of MAGE genes by non-small-cell lung carcinomas. *Int J Cancer* **56**: 826–829

Yamada A, Kataoka A, Shichijo S, Kamura T, Imai Y, Nishida T and Itoh K (1995) Expression of MAGE-1, MAGE-2, MAGE-3/-6 and MAGE-4a/4b genes in ovarian tumours. *Int J Cancer (Pred Oncol)* **64**: 388–393

© Cancer Research Campaign 1998